# Therapeutic potential of vagus nerve stimulation in neurodegenerative diseases: research progress and mechanisms

**DOI:** 10.3389/fimmu.2026.1811107

**Published:** 2026-03-23

**Authors:** Qian Hu, Jiasheng Wang, Ruiyang Cao, Wenkai Liu, Lifeng Wang

**Affiliations:** 1 The First Clinical Medical College of Gannan Medical University, Ganzhou, Jiangxi, China; 2 Department of Anesthesiology, First Affiliated Hospital of Gannan Medical University, Ganzhou, Jiangxi, China; 3 Ganzhou Key Laboratory of Anesthesiology, The First Affiliated Hospital of Gannan Medical University, Ganzhou, Jiangxi, China

**Keywords:** mitochondrial dysfunction, neurodegenerative diseases, neuroinflammation, oxidative stress, vagus nerve stimulation

## Abstract

Neurodegenerative diseases are a group of chronic, progressive neurological disorders caused by the degeneration and functional loss of neurons and glial cells, including Alzheimer’s disease (AD), Parkinson’s disease (PD), Huntington’s disease (HD). Although numerous treatments are available for these diseases, therapeutic outcomes remain unsatisfactory because of their poorly understood pathogeneses of these diseases. Vagus nerve stimulation (VNS), a noninvasive or minimally invasive neuromodulation technique, has shown significant potential in mitigating neurodegenerative conditions. This review explores the mechanisms of action and clinical applications of VNS in neurodegenerative diseases, providing novel insights for the development of novel treatments.

## 1 Introduction

Neurodegenerative diseases (NDDs) are a heterogeneous group of chronic central nervous system disorders characterized by progressive neuronal dysfunction resulting in the gradual loss of neuronal structure/function and myelin degeneration (1). These conditions are becoming increasingly prevalent among elderly individuals and have emerged as leading causes of mortality in this population (2). According to global statistics, stroke (67.4%), Alzheimer’s disease (AD) and other types of dementia (20.3%), and meningitis constitute the three most fatal neurological disorders (3). NDDs are classified on the basis of their primary clinical features, anatomical distribution, or molecular pathology (4), with distinct clinical courses, pathological manifestations, and brain region-specific vulnerabilities (5). The major clinical NDDs subtypes include AD, Parkinson’s disease (PD), dementia with Lewy bodies (DLB), multiple system atrophy (MSA), progressive supranuclear palsy (PSP), and Huntington’s disease (HD), among others (4, 5). AD is the most prevalent neurodegenerative disorder, accounting for 60–70% of all dementia cases worldwide. Current epidemiological data indicate that approximately 32 million individuals are affected by AD, with projections suggesting that disease incidence will double by 2050 (6, 7). The healthcare costs associated with AD are estimated to exceed 2 trillion USD by 2030 (8). These staggering figures underscore the growing global public health challenge posed by NDDs (3).

NDDs typically involve multiple interacting pathological mechanisms that collectively contribute to neural damage and degeneration. While sharing common features, individual NDDs exhibit distinct clinical manifestations and underlying pathogenic mechanisms (5). The main pathogenic mechanisms of various NDDs can be categorized into five types: abnormal protein aggregation and misfolding, neuroinflammation, oxidative stress and mitochondrial dysfunction, genetic and epigenetic factors, and neurotransmitter dysregulation (**Table 1**). Recent clinical interventions are focused primarily on alleviating symptom and slowing disease progression. However, most pharmacological treatments fail to mitigate the underlying pathological processes, and effective strategies to reverse neurodegeneration or restore neurological function are notably lacking, particularly for patients with comorbid conditions (9–12)The vagus nerve, the longest and most extensively distributed cranial nerve in humans, constitutes a critical component of the autonomic nervous system and maintains extensive connections with multiple physiological systems (13). First described as an effective therapy for epilepsy in 1994, VNS has evolved significantly, with its therapeutic applications expanding to various neurological disorders over the last decades (14). Currently, clinically employed VNS approaches primarily involve invasive and noninvasive modalities. Invasive VNS the requires surgical placement of a stimulation device with electrodes coiled around the vagus nerve to deliver continuous stimulation, whereas noninvasive VNS involves the use of transcutaneous or transauricular approaches to stimulate the vagus nerve without surgical intervention. Noninvasive approaches offer distinct advantages, including operational simplicity and increased patient acceptability because of their surgery-free nature (15). Emerging evidence suggests that VNS may have therapeutic effects on various neurological and psychiatric disorders, including epilepsy (16), PD (17), major depressive disorder (18), and schizophrenia (19). Mechanistic studies have indicated that VNS likely exerts neuroprotective effects through multiple convergent pathways, including neuromodulation, anti-inflammatory effects, the reduction of oxidative stress, neurotransmitter regulation, and functional brain network reorganization (20–24). This review systematically discusses the current applications of VNS in NDDs, its potential mechanisms of action, and its therapeutic prospects, thereby providing novel insights for the development of innovative treatment strategies.

**Table 1:** The potential underlying mechanisms associated with NDDs.

Main types of NDDs	Underlying mechanisms associated with NDDs					
	Abnormal protein aggregation and misfolding	Neuroinflammation	Oxidative stress and mitochondrial dysfunction	Neurotransmitter regulation	Genetic factors	Epigenetic factors
Alzheimer’s disease	Aβ plaques and tau protein neurofibrillary tangles	Aβ plaques and neuronal injury release danger signals, such as DAMPs, which activate glial cells to secrete proinflammatory cytokines (e.g., IL-1β, TNF-α) and ROS	Aβ aggregation and hyperphosphorylation of the tau protein lead to calcium overload and excitotoxicity, with an accompanying increase in ROS levels	Ach imbalance	APOEϵ4 increases risk	CLU and PICALM influence lipid metabolism and synaptic function
Parkinson’s disease	α-Synuclein aggregation leading to Lewy body formation	Activated microglia and astrocytes	Interaction between P53 and Parkin; inhibition of mitochondrial complex I; abnormal PINK1–Parkin pathway	Reduced DRD2 activation	Mutations in the SNCA gene; mutations in the LRRK2, Parkin, and PINK1 genes	Histone modifications regulating α-synuclein
Huntington **‘**s disease	mHTT aggregation	Activation of microglia and astrocytes; increase in inflammatory cytokine levels	mHTT disrupts mitochondrial function, the electron transport chain, and calcium homeostasis	Abnormal glutamatergic signaling; loss of synaptic plasticity; damage to the corticostriatal circuit	Repetitive CAG sequences lead to pathogenic mHTT	Histone acetylation/deacetylation imbalances
**Multiple sclerosis**		Increased release of inflammatory mediators by activated microglia	Excessive accumulation of ROS and glutamate during inflammation; excessive astrocyte proliferation		HLA-DRB1*15:01	Methylation changes in immune genes

NDDs, neurodegenerative diseases; Aβ, amyloid-beta; DAMPs, damage-associated molecular patterns; IL-1β, interleukin-1β; TNF-α, tumor necrosis factor-α; ROS, reactive oxygen species; APOEϵ4, apolipoprotein E epsilon 4 allele; CLU, clustering factor; PICALM, phosphatidylinositol-coupled membrane assembly protein; Ach, acetylcholine; P53, cellular tumor antigen p53; PINK1, PTEN-induced putative kinase 1; SNCA, α-synuclein; LRRK2, leucine-rich repeat kinase 2; DRD2, dopamine receptor D2; mHTT, mutant huntingtin protein; CAG, cytosine-adenine-guanine.

## 2 Anatomy and physiology of the vagus nerve

**Figure 1:**
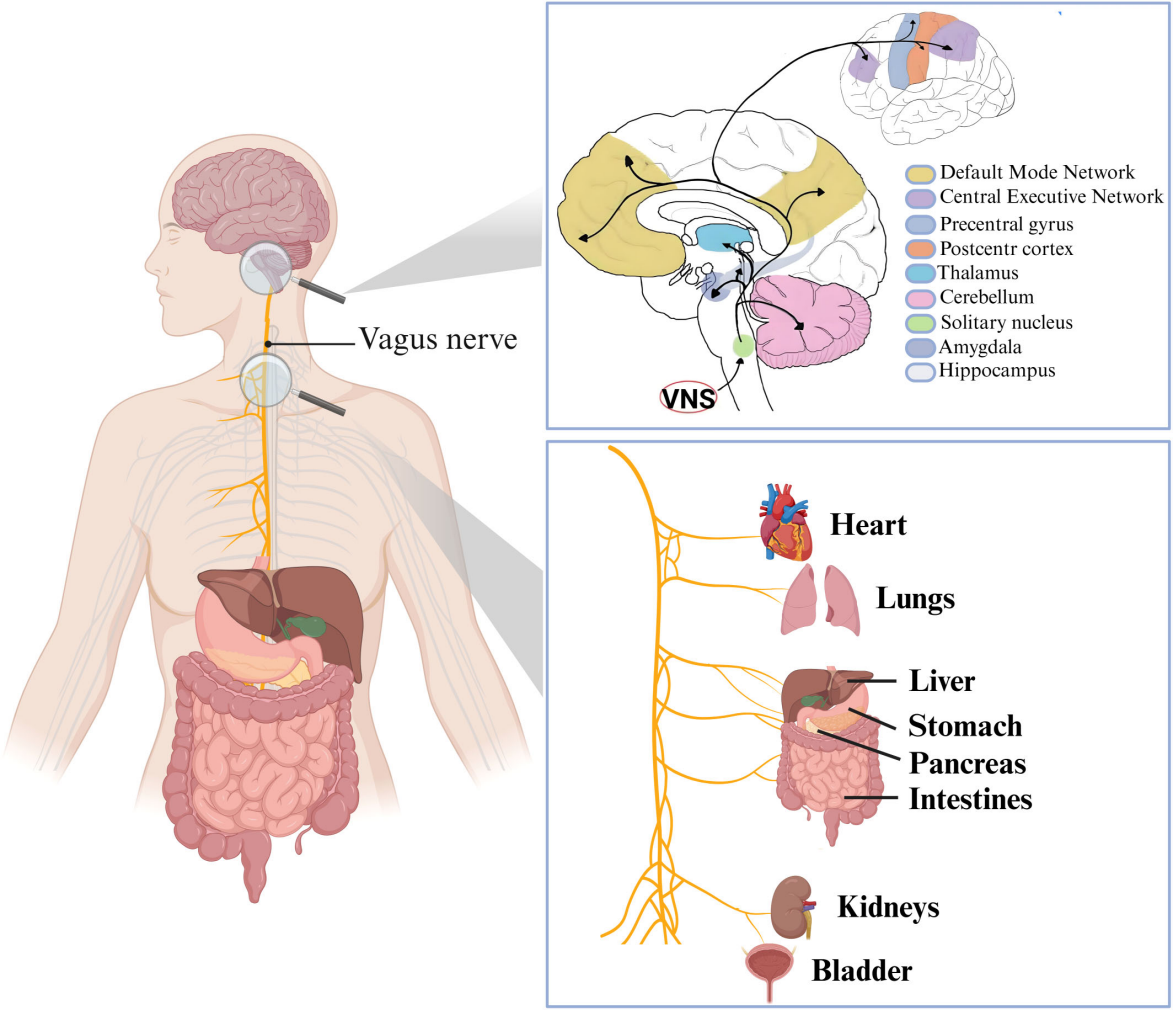
Anatomical diagram of the vagus nerve and its target organ distribution. The diagram depicts the vagus nerve extending through the cervical/thoracic regions to innervate eight core organs: the heart, lungs, liver, stomach, pancreas, intestines, kidneys, and bladder. This diagram visually maps the anatomical pathway of the vagus nerve and its target organ distribution, highlighting its role in the parasympathetic regulation of visceral functions. This structural blueprint serves as a critical anatomical reference for the clinical applications of VNS. VNS: vagus nerve stimulation.

The vagus nerve, the primary parasympathetic nerve and a key component of the autonomic nervous system, is a mixed nerve composed of approximately 80% unmyelinated afferent sensory fibers and 20% myelinated efferent motor fibers (25). Originating from the brainstem, the vagus nerve gives rise to multiple branches that provide extensive innervation to the cervical, thoracic, and abdominal regions (26) (**Figure 1**). Special visceral efferent fibers originating from the nucleus innervate the striated muscles of the larynx and pharynx, whereas general visceral efferent fibers from the dorsal motor nucleus mediate parasympathetic regulation of the cardiac, pulmonary, and gastrointestinal systems through the acetylcholine-mediated activation of muscarinic receptors (M receptors) (27–29). The auricular branch of the vagus nerve (ABVN) represents the only superficial branch of the vagus nerve (30), and forms cutaneous receptive fields primarily distributed in the cymba conchae and cavum conchae of the auricle. The cymba conchae, located on the posterior aspect of the auricle, appears to be the exclusive superficial area in which ABVN fibers are distributed, with evidence suggesting that ABVN terminals are predominantly concentrated in this region (31–33). This anatomical distribution makes the cymba conchae a commonly targeted site for transcutaneous auricular vagus nerve stimulation (taVNS) in clinical studies (34). Characterized by its fine caliber and high sensitivity, the ABVN efficiently transmits electrical stimulation signals, thereby modulating overall vagus nerve activity.

**Figure 2:**
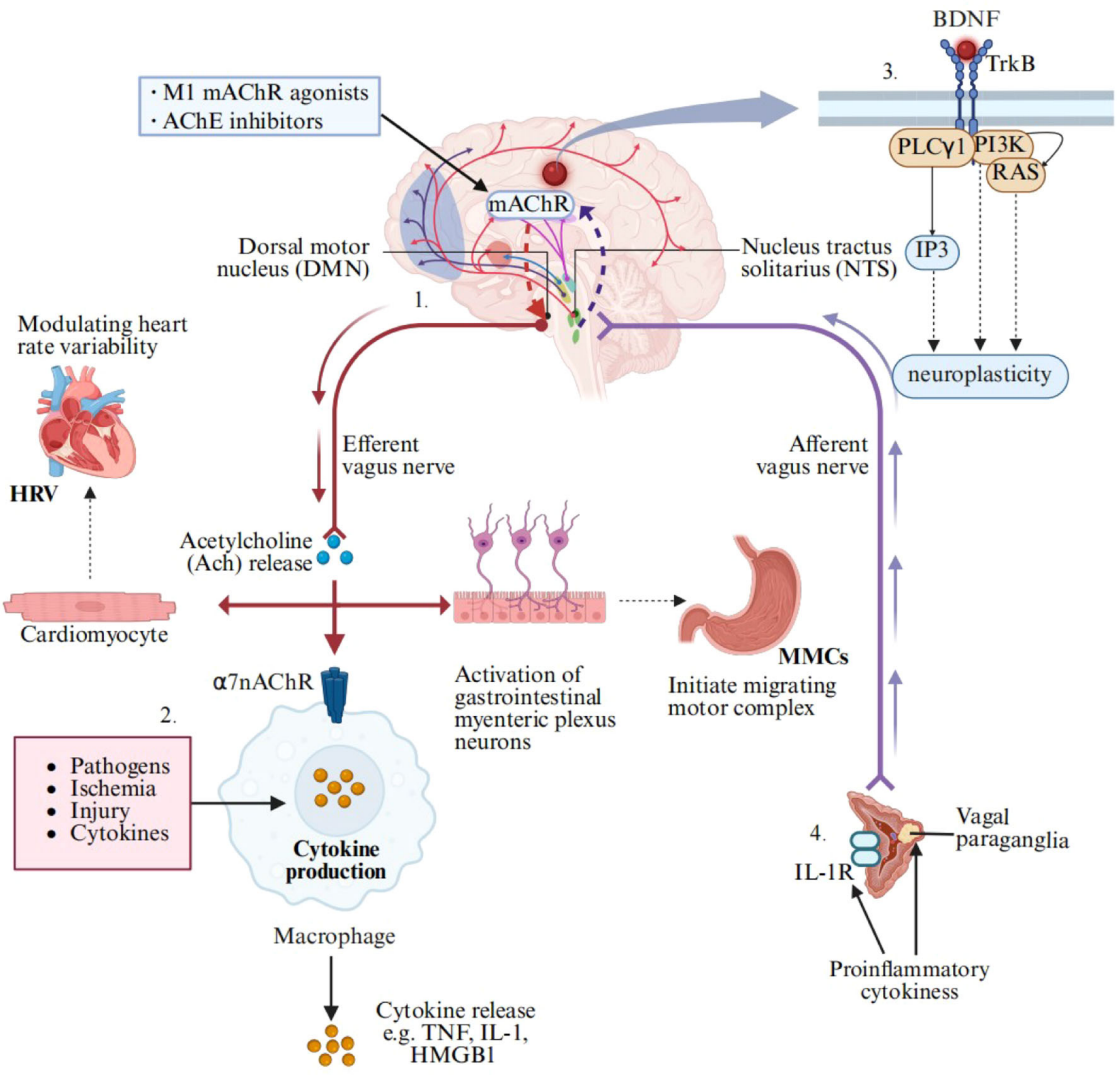
Molecular mechanisms of vagus nerve stimulation. The schematic delineates the mechanisms of vagus nerve stimulation from the molecular to systemic levels: (1) afferent signals are integrate in brainstem nuclei (NTS/DMN), triggering efferent cholinergic output; (2) ACh binds to α7nAChR on macrophages to inhibit the release of proinflammatory cytokines (TNF/IL-1β/HMGB1); (3) neuroplasticity is modulated via the BDNF and PI3K/RAS pathways; (4) synergistic anti-inflammatory and neuroimmunomodulatory effects are exerted. mAChR, muscarinic acetylcholine receptor; M1 mAChR, muscarinic acetylcholine receptor M1 subtype; AChE, acetylcholinesterase; BDNF, brain-derived neurotrophic factor; TrkB, tropomyosin receptor kinase B; PLCγ1, phospholipase Cγ1; IP3, inositol 1, 4, 5-trisphosphate; PI3K, phosphoinositide 3-kinase; RAS, rat sarcoma viral oncogene homologue; HRV, heart rate variability; MMCs, gastrointestinal migrating motor complexes; α7nAChRs, α7 nicotinic acetylcholine receptors; TNF, tumor necrosis factor; IL-1, interleukin-1; HMGB1, high mobility group box 1; IL-1R, interleukin-1 receptor.

The vagus nerve orchestrates multilevel physiological regulatory processes through the “brain–organ axis”. 1. Autonomic Balance Regulation: As the principal parasympathetic pathway, the vagus nerve maintains autonomic homeostasis via cholinergic suppression of sympathetic overactivation, thereby modulating heart rate variability (HRV) and gastrointestinal migrating motor complexes (MMCs) (35, 36). 2. Neuroimmune Modulation: The cholinergic anti-inflammatory pathway (CAP), which is mediated by α7 nicotinic acetylcholine receptors (α7nAChRs), inhibits nuclear factor kappa-B (NF-κB) signaling and significantly downregulates the expression of proinflammatory cytokines (IL-1β, TNF-α) in microglia (37). 3. Central Integration: The nucleus tractus solitarius (NTS) serves as the primary integration center, participating in emotional encoding via the parabrachial–amygdala pathway while regulating sleep-wake cycles through ascending reticular formation projections (38). 4. Neuroplasticity: The vagus nerve exhibits long-term potentiation (LTP)-dependent synaptic reorganization mediated by the brain-derived neurotrophic factor (BDNF)–TrkB signaling cascade, revealing critical molecular targets for neurodegenerative disease intervention (39). These multilayered regulatory mechanisms collectively underscore the pivotal role of the vagus nerve in the brain–organ axis, providing both theoretical insights into physiological homeostasis and translational potential for disease modulation (**Figure 2**).

## 3 Methods of vagus nerve stimulation

**Figure 3:**
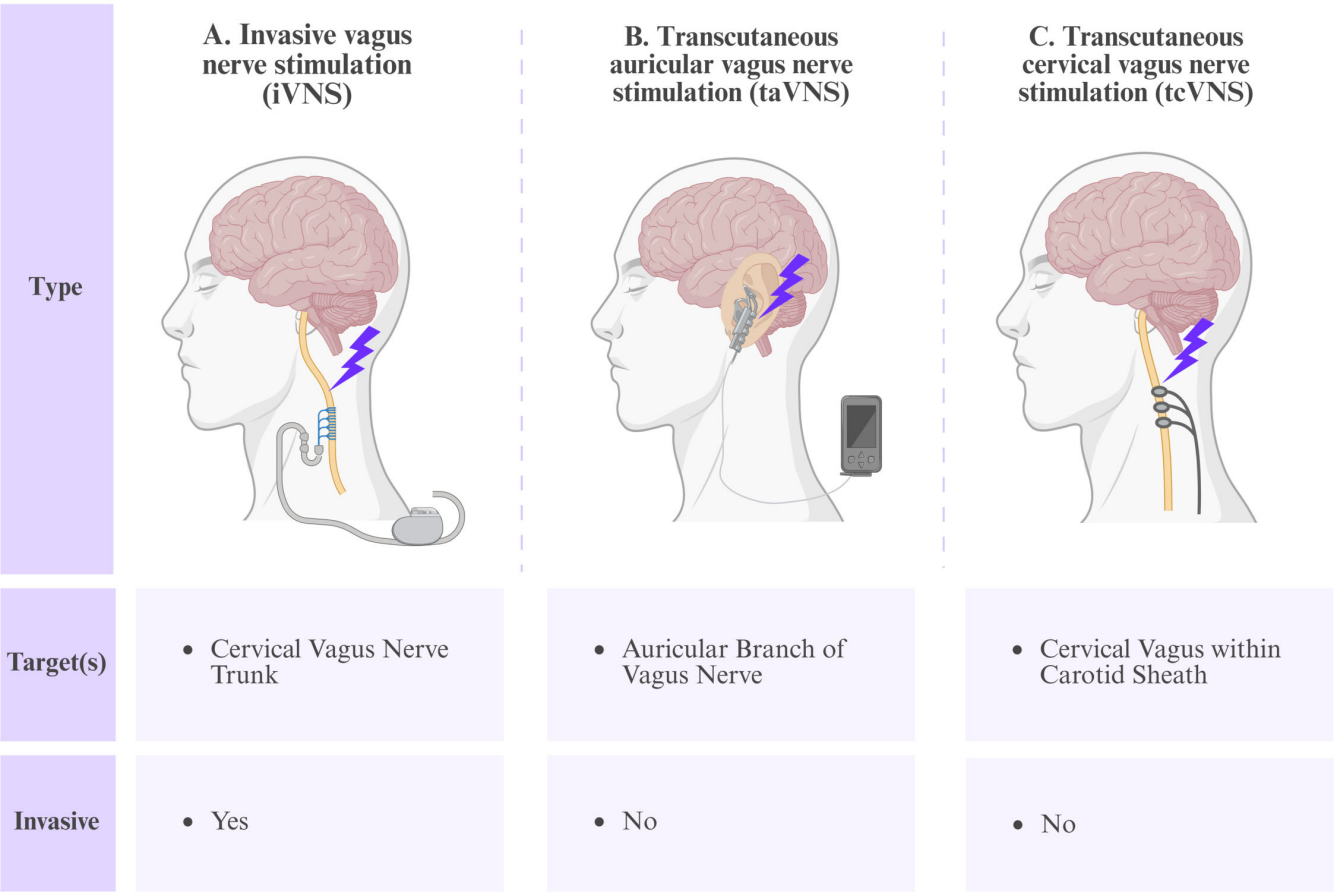
Classification of vagus nerve stimulation techniques. **(A)** iVNS involves the surgical implantation of a neurocybernetic prosthesis encircling the cervical vagus nerve trunk within the carotid sheath, which requires chronic neuromodulation. **(B)** taVNS involves the noninvasive engagement of somatosensory afferents of the auricular branch via cutaneous electrodes positioned at the cymba conchae or tragus, eliciting central neuromodulatory effects through polysynaptic projections to the nucleus tractus solitaire. **(C)** tcVNS involves the application of percutaneous electrical stimulation to the cervical vagus nerve trunk at the anterolateral neck, necessitating meticulous anatomical localization to optimize stimulation fidelity while mitigating the off-target activation of adjacent structures.

VNS involves primarily invasive and noninvasive approaches. Invasive VNS (iVNS) requires the surgical placement of a neurostimulation device with helical electrodes coiled around the vagus nerve to deliver chronic intermittent electrical stimulation. In contrast, noninvasive vagus nerve stimulation (niVNS) employs transcutaneous or transauricular stimulation techniques, and offers significant advantages, including minimal procedural complexity and the avoidance of surgical intervention (**Figure 3**).

iVNS is a neuromodulation technique involving surgical implantation. The most common iVNS approach is cervical VNS. This method requires neck surgery in which electrodes are wrapped around the left cervical vagus nerve, and connected via subcutaneous leads to a pulse generator implanted in the chest. The generator delivers electrical pulses to stimulate the vagus nerve at preset intervals or on demand (40). Its mechanism of action involves primarily modulating afferent pathways of the vagus nerve, thereby influencing neural circuits in the cerebral cortex and limbic system to promote neuroplasticity and stabilize neural networks (40–42). iVNS enables precisely targeted stimulation with minimal energy loss, allowing long-term, stable therapy (43). However, surgical risks include complications such as hoarseness and dysphagia, implant-related infections, and battery depletion (44). niVNS involves the delivery of transcutaneous electrical stimulation, eliminating surgical risks. Current niVNS methods include taVNS and transcutaneous cervical VNS (tcVNS). taVNS involves the stimulation of the ABVN through contact with the external ear skin. Sush stimulation activates vagal pathways to modulate brain function via bottom-up systemic neuromodulation, regulating the autonomic nervous system while alleviating anxiety and improving cognition (45, 46). The compact devices used for taVNS require minimal training and exhibit fewer side effects (47), resulting in excellent tolerability and compliance (46). tcVNS involves the stimulation of the cervical vagal fibers to influence brain and organ function (15). By targeting vagal fibers within the carotid sheath, tcVNS modulates the activity of brainstem nuclei, the autonomic nervous system, and associated brain regions, showing therapeutic potential for depression, epilepsy, migraine, and inflammatory bowel disease (48, 49).

## 4 Mechanisms of the effects of VNS on neurodegenerative diseases

VNS is a therapeutic intervention that involves the delivery of electrical pulses to the vagus nerve via implanted devices, modulating brain activity to treat neurological conditions (50). This stimulation influences both visceral organs innervated by the vagus nerve and central neural functions, thereby producing therapeutic effects. VNS may affect the pathogenesis and progression of NDDs through multiple pathways. Although the precise underlying mechanisms remain incompletely understood, current evidence suggests that VNS ameliorates symptoms by regulating neurotransmitter systems, reducing protein misfolding and aggregation, mitigating neuroinflammation, attenuating oxidative stress and mitochondrial dysfunction, and modulating genetic/epigenetic factors (51, 52). Overall, these findings indicate that VNS exerts multifaceted effects on NDDs through structural and functional neuronal reorganization.

### 4.1 Regulation of neurotransmitters

When dysregulated, neurotransmitters, which serve as endogenous chemical messengers for signal transmission across synapses (between neurons) and neuromuscular junctions, play central roles in the pathogenesis and progression of NDDs (53). In the central nervous system, neurons are classified into the cholinergic, monoaminergic, glutamatergic, or GABAergic subtypes on the basis of their primary neurotransmitters. The cholinergic system is critically involved in key physiological processes, including attention, learning, memory, the stress response, arousal, sleep regulation, and sensory information processing (54). In AD, severe degeneration of cholinergic neurons in the brain substantially reduces acetylcholine (ACh) synthesis and release. This disruption of normal neurotransmission manifests as cognitive impairment and memory loss (55). PD primarily results from the progressive degeneration of dopaminergic neurons in the substantia nigra pars compacta, leading to marked dopamine (DA) deficiency which causes motor dysfunctions, including resting tremors and bradykinesia (56). Furthermore, imbalances in neurotransmitters such as γ-aminobutyric acid (GABA) and glutamate contribute to multiple NDDs by increasing neuronal excitability and inducing neurotoxicity (57).

VNS may ameliorate neurodegenerative disorders by modulating neurotransmitter release and enhancing neuronal activity and synaptic plasticity within neural circuits (58, 59). Mechanistically, VNS stimulates auricular vagus nerve branches to activate brainstem nuclei, particularly the locus coeruleus and raphe nuclei, thereby promoting the release of norepinephrine (NE) and serotonin (5-HT). Sush neuromodulation regulates cortical arousal, increases neuronal excitability, and improves information processing capacity (26, 60). Concurrently, VNS activates the cholinergic anti-inflammatory pathway to facilitate ACh release, which suppresses neuroinflammation and mitigates neuronal damage (61). Notably, studies have confirmed that VNS-mediated ACh release activates cholinergic systems, strengthens synaptic connectivity, and improves memory and learning, ultimately improving the cognitive performance of AD patients (62, 63). Furthermore, VNS modulates DA synthesis and release to regulate cerebral activity, thereby improving motor function in PD patients (64, 65). Martín-Bastida et al. reported that continuous left cervical VNS in a rat PD model prevented the loss of dopaminergic neurons in the nigrostriatal pathway while increasing the density of dopamine β-hydroxylase (DβH) in the locus coeruleus, consequently ameliorating motor deficits (66). VNS also alleviates nonmotor PD symptoms such as depression and sleep disorders through the neuromodulation of NE and 5-HT, improving patient quality of life (64, 67). Multiple sclerosis (MS), a chronic autoimmune, inflammatory, and neurodegenerative disorder affecting the central nervous system (CNS) (68), frequently presents with fatigue, neurocognitive impairment, and motor dysfunction. VNS improves working memory under sleep-deprived conditions by modulating phasic NE release, potentially maintaining optimal arousal levels for cognitive performance (62, 69). Moreover, taVNS increases cerebral NE concentrations, which critically improves attention, executive function, memory, and emotional recognition (70). Overall, these findings indicate that VNS exerts multiple regulatory effects on neurotransmitters that modify disease progression by ameliorating motor, cognitive, and sleep disturbances.

### 4.2 Reducing protein misfolding and aggregation

Abnormal protein aggregation and misfolding constitute key pathological bases for neuronal decompensation and apoptosis. In the brains of NDD patients, neurotic plaques formed by aberrant Aβ deposition and neurofibrillary tangles resulting from hyperphosphorylated tau protein accumulation drive neurodegenerative changes, functional impairment, and neuronal death, ultimately leading to cognitive deficits (71–73). The pathological features of PD are closely associated with the formation of Lewy bodies by conformationally altered α-synuclein (74). Aggregated α-synuclein disrupts intracellular homeostasis in dopaminergic neurons, inducing oxidative stress, mitochondrial dysfunction, and neuroinflammatory cascades. This process ultimately impairs the nigrostriatal system, resulting in bradykinesia, muscle rigidity, and resting tremors (75).

Recent research on the effects of VNS on abnormal protein aggregation and misfolding remains exploratory. VNS may modulate protein folding and aggregation processes by regulating neurotransmitter release and influencing intracellular signaling pathways. In AD, VNS potentially mitigates Aβ deposition and aberrant tau phosphorylation by increasing synaptic transmission, thereby alleviating disease progression and improving cognitive function (76, 77). In PD patients, VNS may inhibit pathological α-synuclein misfolding and propagation by modulating neuronal activity through neurotransmitter regulation, consequently mitigating symptoms such as tremors and rigidity (21, 78–80).

### 4.3 Mitigating neuroinflammation

NDDs are frequently accompanied by inflammatory responses, which may further exacerbate neuronal damage (81). The abnormal aggregation of β-amyloid (Aβ) and hyperphosphorylation of the tau protein in the brain activate microglia and astrocytes, triggering chronic inflammation. Activated immune cells release proinflammatory cytokines such as interleukin-1β (IL-1β), interleukin-6 (IL-6), and tumor necrosis factor-α (TNF-α). These cytokines not only disrupt interneuronal signaling but also induce oxidative stress, damaging neuronal cell membranes, proteins, and DNA. This cascade ultimately leads to neuronal apoptosis and further aggravates cognitive impairment (82). Significant inflammatory cell infiltration and persistent microglial activation occur in the nigrostriatal regions of PD patients. Released inflammatory mediators not only directly damage dopaminergic neurons but also compromise the integrity of the blood–brain barrier. This damage allows peripheral immune cells and neurotoxic substances to infiltrate the brain more readily, exacerbating the neuroinflammatory microenvironment and accelerating the progressive degeneration of dopaminergic neurons. Consequently, motor symptoms, including bradykinesia and tremors, occur (83, 84).

VNS may attenuate neurodegenerative damage by modulating immune system function and mitigating inflammatory responses (37, 85). The noradrenergic locus coeruleus–norepinephrine (LC-NA) system, which is among the earliest brain regions affected in NDDs, including AD and PD, represents a crucial therapeutic target for these conditions (86, 87). Giorgi et al. reported that the LC-NA pathway maintains neural homeostasis by regulating microglial and astrocyte activation while controlling the release of inflammatory mediators (88). Importantly, VNS exerts its primary effects by modulating the LC-NA pathway, potentially activating anti-inflammatory mediators, including peroxisome proliferator-activated receptor γ (PPARγ) and heat shock protein 70 (HSP70), to maintain cellular quiescence and suppress neuroinflammation (89, 90). VNS exerts its anti-inflammatory effects through three distinct neuroimmunological mechanisms. Primarily, VNS increases the release of norepinephrine (NA), which inhibits NF-κB activation, subsequently downregulating major histocompatibility complex class II (MHC-II) expression in astrocytes and suppressing the production of proinflammatory enzymes, including inducible nitric oxide synthase (iNOS) and cyclooxygenase-2 (COX-2) (91–93). Furthermore, the ACh released from vagal efferent fibers binds to α7nAChRs on macrophages, thereby inhibiting the NF-κB signaling pathway and reducing the release of proinflammatory cytokines such as TNF-α and IL-1β (94). Additionally, VNS modulates mast cell degranulation, alleviating blood–brain barrier and intestinal barrier damage following cerebral ischaemia–reperfusion while attenuating the systemic inflammatory response (95). These coordinated mechanisms collectively maintain neural homeostasis and functional stability, ultimately ameliorating neurodegenerative pathology.

### 4.4 Attenuating oxidative stress and mitochondrial dysfunction

Oxidative stress arises from an imbalance between the excessive accumulation of reactive oxygen species (ROS) and compromised antioxidant defense systems, causing damage to cellular structures, proteins, lipids, and genetic material and accelerating neuronal death (96). Mitochondria, which serve as central hubs for cellular energy metabolism and ROS generation, develop functional impairments that exacerbate bioenergetic deficits and oxidative damage, establishing a self-perpetuating pathological cycle (97). In AD, aberrant Aβ deposition induces excessive ROS production in neurons, triggering oxidative stress. The resulting surge in ROS levels enables ROS to attack mitochondrial membranes, inducing lipid peroxidation that disrupts membrane integrity and fluidity. This process impairs respiratory chain complexes, obstructs electron transport, reduces ATP synthesis, and ultimately causes mitochondrial dysfunction (98). Similarly, in patients with PD, pathological α-synuclein aggregation disrupts mitochondrial physiology, promoting further ROS generation, which exacerbates oxidative stress and amplifies mitochondrial damage, culminating in dopaminergic neuronal degeneration (99).

VNS has beneficial effects on ameliorating oxidative stress and mitochondrial dysfunction. The mitochondrial pathway of cell death is widely recognized to be mediated by the opening of the permeability transition pore (PTP). VNS may stabilize mitochondrial function by inhibiting PTP opening (100, 101), which also ameliorates contractile dysfunction during hypoxia–reoxygenation injury (102). Studies indicate that VNS enhances the body’s antioxidant defense system by increasing the expression and activity of antioxidant enzymes such as superoxide dismutase (SOD) and glutathione peroxidase (GSH-PX), thereby accelerating the clearance of excess ROS and mitigating cellular damage (103, 104). Additionally, VNS modulates mitochondrial function by regulating the expression of mitochondria-related genes and proteins, increasing ATP synthesis capacity, and strengthening neuronal antioxidant defenses. This process reduces oxygen free radical generation, alleviates neuronal oxidative stress, and slows the progression of neurodegeneration (105). VNS has been shown to improve oxidative stress biomarkers in patients with epilepsy and depression, suggesting its potential applicability for treating NDDs (106).

VNS may exert therapeutic effects on various NDDs through the aforementioned mechanisms. In patients with AD, VNS potentially mitigates disease progression by reducing cerebral oxidative stress, improving mitochondrial function, inhibiting Aβ neurotoxicity, and decreasing neuronal damage and death, thereby enhancing cognitive functions, including memory, attention, and executive function (107, 108). In patients with PD, VNS may modulate redox balance, preserve mitochondrial integrity, reduce abnormal α-synuclein aggregation, and protect dopaminergic neurons, all of which could alleviate both typical and atypical motor symptoms (109). Additionally, in patients with amyotrophic lateral sclerosis (ALS), VNS might ameliorate oxidative stress and mitochondrial dysfunction, thereby reducing motor neuron degeneration, increasing neuronal survival, and improving quality of life (110). Future research should focus on elucidating the disease-specific pathways associated with the effects of VNS, particularly the clinical potential of taVNS.

### 4.5 Modulating genetic/epigenetic factors

NDDs are strongly associated with genetic variations and epigenetic dysregulation. In AD, interdependent genetic loci are correlated with tau hyperphosphorylation, whereas aberrant DNA methylation, histone modifications, and noncoding RNA-mediated gene regulation influence neuroinflammation, oxidative stress, and synaptic plasticity. These alterations disrupt neuronal function and accelerate disease progression (111, 112). Specific mutations in genes such as PARK, PINK1, and Parkin drive α-synuclein accumulation, directly inducing neuronal damage in PD patients (113). Abnormal DNA methylation further impairs dopaminergic neuron function by inducing gene expression dysregulation, resulting in bradykinesia and tremors (114).

Experimental studies have revealed that VNS enhances the preference of male rats for novel objects while modulating epigenetic transcriptomes in the hippocampus, cortex, and blood. This intervention regulates genes involved in neuronal plasticity and the stress response (115). Furthermore, VNS potentially upregulates the expression of brain-derived neurotrophic factor (BDNF) to modulate synaptic gene expression, thereby promoting synaptic plasticity (50). Collectively, these mechanisms suggest that VNS may partially reverse genetic and epigenetic dysregulation in individuals with different neurodegenerative pathologies.

## 5 Clinical applications of VNS in NDDs

As an important neuromodulation technique, VNS has achieved significant progress in the clinical translation for NDDs in recent years. Among the various VNS modalities, taVNS has garnered considerable attention in current clinical translation research due to its advantages of being non-invasive, portable, and cost-effective (**Table 2**). The core focus of current VNS clinical translation centers on safety validation, efficacy verification, and clinical application potential, with the translational process exhibiting distinct characteristics across different types of NDDs.

**Table 2:** Disease−based summary of VNS mechanisms and clinical evidence in NDDs.

Main types of NDDs	Key mechanisms involved	Mechanistic evidence	Clinical study summary	Clinical significance	Limitations	References
Alzheimer’s disease	1 Neurotransmitter regulation (Ach, NE, NGF) 2 Neuroinflammation (microglial activation, IL-1β, TNF-α, α7nAChR pathway) 3 Protein aggregation (Aβ plaques and tau protein) 4 Oxidative stress & mitochondrial dysfunction (ROS) 5 Genetic/epigenetic modulation (APOEϵ4, BDNF)	1 One SR, clinical studies, and animal models for neurotransmitter regulation (↑cholinergic) 2 One SR and animal models for neuroinflammation (↓IL−1β, IL-18, α7nAChR pathway) 3 Animal models for protein aggregation (↓Aβ plaques) 4 Animal models for oxidative stress↓ 5 Animal models for genetic/epigenetic (↑BDNF)	1 2RCT (n=52, 30) with taVNS: improvement in cognitive performance. 2 One RCT (n=50) with tVNS: improvement in functional connectivity 3 One MA with VNS: improvement in cognitive function 4 2 pilot study (n=10, 17) with VNS: improved ADAS-cog, MMSE scores, and mild cognitive impairment 5 One registered clinical trial protocol (n=40): safety and feasibility of taVNS	Moderate (positive RCTs, but small sample sizes; need larger confirmatory trials)	1 Limited number of RCTs 2 Short follow-up 3 Fower direct protein aggregation biomarkers 4 No studies measuring APOEϵ4 directly	(93, 120, 121, 130–139)
Parkinson’s disease	1 Neurotransmitter regulation (Dopamine, NE, 5-HT, NGF) 2 Neuroinflammation (microglial activation) 3 Protein aggregation (α-synuclein) 4 Oxidative stress & mitochondrial dysfunction 5 Genetic/epigenetic modulation	1 Clinical studies and animal models for neurotransmitter regulation (↑Dopamine, DA, LC-NA pathway) 2 One SR (22 studies on parkinsonism) for enhance synaptic plasticity and regulate neurotransmitter activity 3 Animal models for neuroinflammation (↓IL-1β, TNF-α, α7nAChR pathway, inflammatory glial cells) 4 Animal models for protein aggregation (↓Aβ plaques) 5 Animal models for oxidative stress	1 One MA (n=217) with nVNS: improvement in freezing of gait 2 One MA (n=176) with tVNS: moderate therapeutic effects on motor functions 3 One SR (n=287) with tVNS: significantly improved gait characteristics 4 2RCT (n=22, 30) with taVNS: relieve gait impairments and remodel sensorimotor integration 5 One RCT (n=33) with tcVNS: minor improvement in gait and cognitive function 6 One pilot study (n=10) with taVNS: improvement of TUG time, speed, and variability	Moderate (positive RCTs, but small sample sizes; need larger confirmatory trials)	1 Heterogeneous stimulation protocols 2 No significant changes in motor functions and most cognitive measures, individual participants showed clinically meaningful improvements 3 fatigue were negatively impacted by tVNS 4 Limited data on protein aggregation 5 Few studies on epigenetic changes	(65, 78, 116–119, 140–147)
Huntington’s disease	1 Neurotransmitter regulation (glutamate, GABA, NGF) 2 Neuroinflammation (microglial activation) 3 Protein aggregation (mHTT) 4 Oxidative stress & mitochondrial dysfunction 5 Genetic (CAG repeats)	1 Animal models for neurotransmitter regulation (↓glutamate) 2 One review for neuroinflammation (restore microbiota-gut-brain-axis homeostasis) 3 One review for neurotransmitter regulation (NGF)	No clinical evidence currently supports the efficacy of VNS for HD	Exploratory	1 Complete lack of human studies 2 The mechanism of action of VNS in HD remains incompletely understood 3 Translational gap from animal models	(147–150)
Multiple sclerosis	1 Neurotransmitter regulation (NE, 5-HT) 2 Neuroinflammation (autoimmune modulation) 3 Oxidative stress & mitochondrial dysfunction	1 Animal models for neurotransmitter regulation (↓glutamate) 2 Animal models for neuroinflammation (↓infiltration of neutrophils and pathogenic lymphocytes, myelin damage, blood–brain barrier disruption, fibrinogen deposition, astrocytic activation, and microglial activation	1 One clinical study(n=3) with VNS: improves cerebellar tremor and dysphagia in multiple sclerosis 2 One registered clinical trial protocol (n=60): targeting non-motor symptoms	Weak (only one small study)	1 Minimal clinical data 2 Lack of trials targeting Multiple sclerosis specific outcomes 3 Unclear optimal stimulation parameters	(110, 122, 123, 151)

Clinical evidence strength integrates findings from mechanistic studies, clinical trials, and systematic reviews/meta-analyses.

Strong: At least one high-quality double-blind, randomised sham-controlled trial demonstrates clinically meaningful benefits, supported by a consistent systematic review/meta-analysis and robust mechanistic evidence.

Moderate: At least one positive RCT (with limitations) or multiple consistent open-label studies exist, with a systematic review suggesting potential efficacy and moderate mechanistic support.

Weak: Clinical data are limited, inconsistent, or derived only from small case series; systematic reviews cannot draw firm conclusions; mechanistic support is preliminary or conflicting.

Exploratory: No clinical studies are available, or only isolated case reports exist; the mechanism is theoretical or based on indirect evidence; systematic reviews find no eligible studies. This level indicates that the field is in its infancy, requiring foundational mechanistic and clinical investigation. This is an internal criterion specific to this review to standardize evidence quality assessment.

NDDs, neurodegenerative diseases; AD, Alzheimer’s disease; PD, Parkinson’s disease; HD, Huntington’s disease; MS, multiple sclerosis; VNS, vagus nerve stimulation; tVNS, transcutaneous vagus nerve stimulation; taVNS, transcutaneous auricular vagus nerve stimulation; nVNS, non-invasive vagus nerve stimulation; tcVNS, transcutaneous cervical vagus nerve stimulation; RCT, randomized controlled trial; SR, systematic review; MA, meta-analysis; ACh, acetylcholine; NE, norepinephrine; NGF, nerve growth factor; 5-HT, 5-hydroxytryptamine; DA, dopamine; GABA, gamma-aminobutyric acid; IL-1β, interleukin-1β; IL-18, interleukin-18; TNF-α, tumor necrosis factor-α; α7nAChR, alpha7 nicotinic acetylcholine receptor; Aβ, amyloid-beta; α-synuclein, alpha-synuclein; mHTT, mutant huntingtin; ROS, reactive oxygen species; APOEϵ4, apolipoprotein E epsilon 4; BDNF, brain-derived neurotrophic factor; LC-NA, locus coeruleus-noradrenergic; CAG, cytosine-adenine-guanine; ADAS-cog, Alzheimer’s Disease Assessment Scale-Cognitive; MMSE, Mini-Mental State Examination; TUG, Timed Up and Go test; BBB, blood–brain barrier. n, Number in the study. ↑, Increase. ↓, Reduce.

VNS has been most extensively studied in the treatment of PD, with its efficacy and safety well established. A 2025 systematic review and meta-analysis encompassing 12 studies with a total of 287 participants demonstrated that non-invasive VNS improves gait characteristics in PD patients, including stride length, gait speed, and gait flexibility, with favorable safety and tolerability profiles (116). Similarly, a study by Shan et al., which included 6 randomized controlled trials involving 176 PD patients, revealed that tVNS administered during medication periods improved motor function (severity of motor signs -0.48 [95% CI -0.93, -0.04], gait -0.48 [95% CI -0.85, -0.1], patients reported non-motor outcomes -0.4 [95% CI -0.78, -0.03]), with no definitive adverse events reported (117). Abouelmagd ME et al. also noted that adverse effects associated with taVNS were predominantly mild and transient, with no serious adverse events directly related to stimulation parameters (118). As a potential alternative to invasive VNS, non-invasive taVNS has demonstrated relatively consistent clinical benefits in PD (119). Furthermore, the mechanisms underlying VNS efficacy in PD involve multiple pathways, including modulation of the gut microbiota-gut-brain axis, inhibition of central and peripheral neuroinflammation, and enhancement of neurotrophic factor secretion.

In AD, the core value of VNS application lies in improving cognitive function. A double-blind randomized controlled trial demonstrated that 24 weeks of taVNS intervention improved scores on multiple cognitive measures, including the Montreal Cognitive Assessment-Basic, Auditory Verbal Learning Test, and Boston Naming Test, with only mild and reversible adverse effects, confirming its clinical safety and efficacy (120). The ongoing VINCI-AD.

Trial (Safety and feasibility of transcutaneous vagus nerve stimulation in mild cognitive impairment) will further evaluate the safety and feasibility of taVNS in 40 participants, providing additional evidence for early intervention in AD (121). For HD and MS, clinical evidence for VNS remains extremely limited. Currently, no clinical studies support the efficacy of VNS in treating HD. In the MS field, animal experiments have confirmed that VNS can ameliorate inflammatory responses and promote remyelination (110). However, only one small-scale clinical study has shown that VNS improves cerebellar tremor and dysphagia (122), and one registered clinical trial is currently exploring its effects on non-motor symptoms (123).

In summary, current VNS research predominantly focuses on the treatment of PD. Improvements observed in other NDDs are primarily derived from animal experiments, and clinical translation for these conditions requires further support from additional clinical studies, particularly high-quality randomized controlled trials or multicenter studies.

## 6 Discussion

As a neuromodulation technique, VNS has considerable potential for treating NDDs. VNS ameliorates symptoms such as cognitive and motor impairments by improving functional connectivity within neural networks (124–127). For instance, electrical stimulation at specific acupoints significantly improves cognitive function and alleviates comorbid affective disorders (e.g. anxiety and depression), thereby delaying disease progression in patients with conditions such as PD and AD (128, 129). However, the clinical translation of VNS faces several significant challenges. First, the mechanistic bases of its therapeutic effects, particularly those of noninvasive VNS, remain incompletely understood, impeding the rational optimization of treatment protocols. Second, there is a notable lack of standardized VNS protocols, as stimulation parameters (frequency, intensity, and duration) vary considerably across studies, complicating the comparison of outcomes and the establishment of clinical guidelines. Third, substantial interindividual variability in treatment response, influenced by factors such as neuroanatomical variations, disease progression, and comorbid conditions, complicates the prediction of therapeutic efficacy. Safety and tolerability also present concerns: iVNS is associated with carries inherent surgical risks (e.g., infection, and nerve injury), whereas noninvasive methods may cause local skin irritation or discomfort. Furthermore, while evidence for the ability of VNS to treat epilepsy and depression is more established, its disease-specific efficacy across the spectrum of NDDs remains an emerging area of research, necessitating targeted investigations.

Given these challenges, many unknowns persist regarding the optimal therapeutic application of VNS for NDDs. Future research must prioritize elucidating the precise pathophysiological mechanisms through which VNS exerts its effects on specific NDDs, which will provide a crucial theoretical foundation for refining intervention strategies. Furthermore, large-scale, multicenter, long-term clinical trials are essential to establish optimized, standardized stimulation parameters and to comprehensively evaluate long-term efficacy and safety. As mechanistic understanding deepens and clinical methodologies improve, VNS is anticipated to evolve into a more refined and accessible therapeutic option, offering better alternatives for patients with NDDs.
